# Absence of myeloid Klf4 reduces prostate cancer growth with pro-atherosclerotic activation of tumor myeloid cells and infiltration of CD8 T cells

**DOI:** 10.1371/journal.pone.0191188

**Published:** 2018-01-11

**Authors:** David J. Barakat, Rahul Suresh, Theresa Barberi, Kenneth J. Pienta, Brian W. Simons, Alan D. Friedman

**Affiliations:** 1 Department of Oncology, Johns Hopkins University School of Medicine, Baltimore, Maryland, United States of America; 2 Department of Urology, Johns Hopkins University School of Medicine, Baltimore, Maryland, United States of America; University of South Alabama Mitchell Cancer Institute, UNITED STATES

## Abstract

The microenvironment of prostate cancer often includes abundant tumor-associated macrophages (TAMs), with their acquisition of an M2 phenotype correlating with local aggressiveness and metastasis. Tumor-derived M-CSF contributes to TAM M2 polarization, and M-CSF receptor inhibition slows prostate cancer growth in model systems. As additional cytokines can direct TAM M2 polarization, targeting downstream transcription factors could avoid resistance. Klf4 and C/EBPβ each contribute to monocyte development, and reduced expression of macrophage Klf4 or C/EBPβ favors their adoption of a pro-inflammatory M1 state. We find that a Hi-Myc C57BL/6 prostate cancer line grows more slowly in syngeneic Klf4(f/f);Lys-Cre compared with Klf4(f/f) mice when inoculated subcutaneously, but grows equally rapidly in C/EBPβ(f/f);Lys-Cre and C/EBPβ(f/f) hosts. In the absence of myeloid Klf4, TAMs have reduced expression of surface mannose receptor and *Fizz1* mRNA, both M2 markers. Global gene expression analysis further revealed activation of pro-inflammatory, pro-atherosclerotic pathways. Analysis of tumor-infiltrating lymphocytes (TILs) demonstrated markedly increased activated CD8 T cell numbers, and CD8 T cell depletion obviated the inhibitory effect of myeloid Klf4 deletion on prostate cancer growth. These findings suggest that reducing expression or activity of the Klf4 transcription factor in tumor myeloid cells may contribute to prostate cancer therapy.

## Introduction

The micro-environment of prostate cancer (PCa) often includes abundant tumor-associated macrophages (TAMs). In a study of 131 PCa patients, increased TAMs correlated with PSA >50 and increasing Gleason score or T stage. In a multivariate analysis of these data that included PSA, Gleason score, extra-capsular extension, lymph nodes metastasis, and distant metastasis, increased TAMs was an independent poor prognostic factor for recurrence-free survival, with a hazard ratio of 2.7 [1].

Extra-cellular signals direct macrophages to a range of gene expression patterns, including the pro-inflammatory M1 and the alternatively-activated M2 states, with the majority of macrophages in a variety of established malignancies, including PCa, assuming the M2 phenotype [2–5]. Metastatic PCa lesions have increased mannose receptor (MR)-expressing M2 TAMs compared with adjacent osseous tissue [6]. Tumors resected from 93 non-metastatic PCa patients were evaluated for M1 and M2 TAM polarization. Those with extra-capsular extension (mainly Gleason 9, T3) demonstrated, on average, 4-fold more scavenger receptor expressing M2 versus scavenger receptor negative M1 TAMs, whereas organ-confined tumors (Gleason 6–7, T2) had 1.5-fold more M1 compared with M2 TAMs [7]. Moreover, in the latter study, increased M2 TAMs was associated with more rapid biochemical recurrence, in both the entire population and the subset with extra-capsular extension.

Elimination of M2 TAMs, or their conversion to the anti-tumor M1 phenotype, has therapeutic potential. Colony-stimulating factor 1 receptor (CSF1R) tyrosine kinase inhibition reduces murine PCa TAMs 15-fold, lowers expression of the *Vegfa*, *Mmp9*, and *Arg1* M2 mRNAs in the remaining TAMs, and delays tumor progression, with similar findings in immune-deficient mice inoculated with a human PCa line [8]. However, resistance to CSF1R-targeted therapy might arise via tumor secretion of alternative M2-polarizing cytokines, such as IL-4, as seen in a glioma model [9]. Targeting transcription factors that mediate M2 TAM polarization downstream of multiple cytokines might by-pass these resistance mechanisms.

Transcription factors contributing to M2 macrophage polarization include STAT6, PPARγ, NF-κB p50, Klf4, and C/EBPβ. STAT6 and PPARγ are induced by IL-4 and cooperate in M2 gene activation, while absence of the inhibitory NF-κB p50 subunit favors activation of pro-inflammatory NF-κB p65 target genes [10]. Klf4 and C/EBPβ each play a role in monocytic maturation. Increased levels of the PU.1 transcription factor favors monopoiesis over granulopoiesis and PU.1 activates *Klf4* transcription, likely in cooperation with the PU.1 partner IRF8. Klf4 rescues monopoiesis in the absence of PU.1; absence of Klf4 in marrow progenitors reduces monopoiesis, whereas exogenous Klf4 increases monopoiesis [11–15]. C/EBPβ is a leucine zipper transcription factor that binds C/EBP *cis* elements upon dimerization with another C/EBP family member; in addition, C/EBPβ zippers with AP-1 proteins such as JunB or c-Fos to bind composite DNA elements [16]. Such hybrid elements are found in regulatory regions of monocytic genes, and exogenous C/EBP:AP-1 heterodimers, but not C/EBP or AP-1 complexes alone, direct monopoiesis [17, 18].

Murine and human bone marrow-derived macrophages (BMDM) manifest reduced *Klf4* mRNA and protein in response to M1-polarizing lipopolysaccharide (LPS), whereas M2-polarizing IL-4 induces a striking increase in *Klf4* [19]. BMDM from Klf4(f/f);Lys-Cre mice, lacking myeloid Klf4 due to the lineage-restricted activity of the Lysozyme promoter, manifest reduced IL-4 induction of the *Arg1*, *MR*, *Fizz1*, and *Ym1* M2 mRNAs, and enhanced expression of the *Cox2*, *Tnfα*, and *Nos2* M1 mRNAs [19]. We find that a syngeneic Hi-Myc PCa line grows slower in Klf4(f/f);Lys-Cre compared with Klf(f/f) mice, whereas growth was not impaired in mice lacking myeloid C/EBPβ. In the absence of myeloid Klf4, TAMs had reduced expression of surface mannose receptor and *Fizz1*. Although classic M1 markers such as *Nos2* and *Tnfα* were not increased, microarray analysis revealed activation of pathways associated with pro-inflammatory states, as well as the expression of a subset of mRNAs associated with macrophage activation in atherosclerosis. In addition, tumor-infiltrating CD8 T cell numbers were markedly increased and CD8 T cell depletion obviated the slower tumor growth seen in Klf4(f/f);Lys-Cre hosts. These findings suggest that reducing expression or activity of the Klf4 transcription factor in tumor myeloid cells may contribute to prostate cancer therapy.

## Materials and methods

### Mice and ethics statement

Wild-type (WT) C57BL/6 (B6) mice were obtained from Charles River Laboratories. Lysozyme-Cre (Lys-Cre) B6 mice were obtained from Jackson Laboratory (#004781). Klf4(f/f) and C/EBPβ(f/f) B6 mice were obtained from the Mutant Mouse Regional Resource Center (#29877 and #34760). This study was carried out in strict accordance with the recommendations in the Guide for the Care and Use of Laboratory Animals of the National Institutes of Health. The protocol (M013M116) was approved by the Johns Hopkins University Animal Care and Use Committee. All efforts were made to minimize suffering. Euthanasia was carried out by carbon dioxide inhalation followed by verification of death.

### B6 Hi-Myc prostate cancer cells

After back-crossing FVB Hi-Myc mice [20] into the B6 background for >10 generations, a metastatic lesion was identified in a prostate draining lymph node from an 17-month-old mouse. Cells from this lesion were dissociated and passaged serially by subcutaneous (SQ) inoculation into the flanks of B6 mice as a B6 Hi-Myc PCa line. Hematoxylin and eosin staining of paraffin embedded tumors revealed dysplastic epithelial cells, and immunohistochemistry confirmed their expression of c-Myc and AR (S1 Fig) and CK8 (not shown). Androgen-sensitivity was confirmed by the rapid tumor regression evident upon orchiectomy (not shown).

### Tumor cell inoculation

When tumors reached 1.2–1.5 cm, mice were euthanized and tumor tissue was collected, minced with a razor blade, washed with phosphate-buffered saline (PBS), resuspended in 5mL DMEM/F12 media containing 10% FBS, 500 μL Collagenase/Hyaluronidase (Stem Cell Technologies), 2.5U/mL Dispase and 0.05mg/ml Dnase I, and then incubated at 37°C for 1 hr with occasional mixing. Tumor tissue was vigorously triturated and passed through a 40 μM cell strainer with the aid of a syringe plunger. Cells were then pelleted at 350 g x 5 min and resuspended in PBS. Live cells were then enumerated using Trypan Blue dye and a hemocytometer, and 2E6 viable cells in 100 μL PBS were injected SQ into the shaved flank of mice anesthetized with isofluorane. Tumor growth was monitored using caliper measurements of length (L), width (W) and height (H), with volume estimated from the ellipsoid volume formula: *V* = *L* * *W* * *H* * *π*/6 [21]. Mice were euthanized when tumors reached 2.0 cm in largest diameter or volume greater than 1000 mm^3^. Paraffin-embedded tumors were subjected to hematoxylin-eosin staining following by imaging at 400X using a Nikon E400 microscope, with attached CCD camera.

### T cell depletion

To deplete CD8 T cells, mice were inoculated with 200 μg rat-anti-CD8 antibody (Bio X Cell, BE0117) intraperitoneally on days -7, -5, -3 and 0 relative to Hi-Myc PCa cell inoculation (day 0). Peripheral blood obtained on d -1 from mice was subjected to flow cytometry analysis to assess depletion.

### Myeloid and T cell flow cytometry analysis

Tumors were dissociated as for cell inoculation and subjected to flow cytometry (FC) analysis, gating on live cells lacking staining with Live/Dead Aqua (ThermoFischer). All antibody staining was preceded by FcγR block on ice (eBioscience/Fisher). Extracellular antibodies were then added and samples were incubated on ice. Intracellular staining was accomplished after surface staining using the FoxP3 staining kit (eBioscience). To evaluate myeloid subsets, total cells were stained with anti-CD11b-FITC, anti-CD45-BV650, anti-Ly6C-AF700, anti-MR-PE-Cy7, anti-CD11c-PE/Dazzle594, and anti-Ly6G-BV605 (BioLegend); anti-MHCII-eFluor450 (eBioscience); anti-CD86-PE (Miltenyi); and anti-F4/80-APC (BioRad). T cells were enumerated by staining with anti-CD45-AF700, anti-CD3-AF488, anti-CD4-PE, anti-CD8-BV655, and anti-CD69-APC-Cy7 (BioLegend), followed by intracellular stain with anti-IFNγ-APC (BioLegend). To evaluate Tregs, total cells were stained with anti-CD3-AF488, anti-CD4-BV605, and anti-CD25-PerCP-Cy5.5 (BioLegend), and then stained intracellularly with anti-FoxP3-PE (BD Pharmingen).

### RNA analysis

Tumor CD11b^+^ cells were isolated using an anti-CD11b immunomagnetic isolation kit (Miltenyi), and RNA was isolated using NucleoSpin RNA II kit (Machery-Nagel). First-strand cDNA was prepared using AMV reverse transcriptase (Promega) and oligodT primer. Quantitative real-time PCR (qRT-PCR) was carried out using Lo-Rox SYBR Green (Alkali Scientific). Primer pairs are provided (S1 Table). For global gene expression analysis, total RNA was prepared from tumor CD11b^+^ cells using the NucleoSpin RNA II kit. RNA quality was confirmed via Nanodrop-1000 spectrometric analysis and via a Bioanalyzer (Agilent Technologies). 500 ng total RNA from each sample was amplified and labeled using the Illumina TotalPrep RNA Amplification Kit with oligo(dT) priming (Ambion). 750 ng biotin-labeled cRNA was combined with hybridization buffer and hybridized to the Agilent Mouse GE 4 x 44K v2 Microarray at 58°C for 16–20 hours. After hybridization, the array was washed with buffer at 55°C and blocked at room temperature. Bound, biotinylated cRNA was stained with streptavidin-Cy3 and then washed. Dried arrays were scanned with the iScan System, and data with quantile normalization and background correction to 100 was exported from GenomeStudio v2011.1 Gene Expression Module 1.9.0 and imported to GeneSpring. Differentially expressed genes with >1.4-fold mean change were subjected to pathway analysis using Ingenuity Systems software with false discovery rate (FDR) = 0.05 as threshold.

### Immunohistochemistry

Immunohistochemistry was performed as previously described [22]. Briefly, heat-induced antigen retrieval was carried out in a steam chamber followed by washing in Tris-buffered saline. Endogenous peroxidases were inactivated with Bloxall (Vector labs), and sections were blocked with Dako blocking buffer (Agilent Technologies), incubated with primary antibodies (anti-c-Myc, anti-AR), washed, and then incubated with horse radish peroxidase-conjugated secondary antibodies. Chromagen was developed with DAB solution (Vector Labs) and counterstained with Meyer’s hematoxylin. Primary antibodies used were anti-cMyc (Abcam, clone 769, 1:1000), anti-Androgen Receptor (Santa Cruz, clone N-20, 1:500), and anti-CK8 (Covance, clone 1E8, 1:1000).

### Statistics

Tumor volumes, tumor growth rates, myeloid and T cell subsets, and RNA expression values were compared using the Student *t* test. Tumor growth rates were estimated by fitting exponential curves to volume data (Excel) for each tumor according to the equation V = V_o_*e*^bt^ (V = volume, V_o_ = initial volume, t = time in days and b = exponential slope). The slope or growth rate was estimated as the value ‘b’ from the above equation because ln(V) = ln(V_o_) + bt. Means and standard errors (SE) are shown.

## Results

### Hi-Myc prostate cancer growth is slowed in the absence of myeloid Klf4

**Fig 1:**
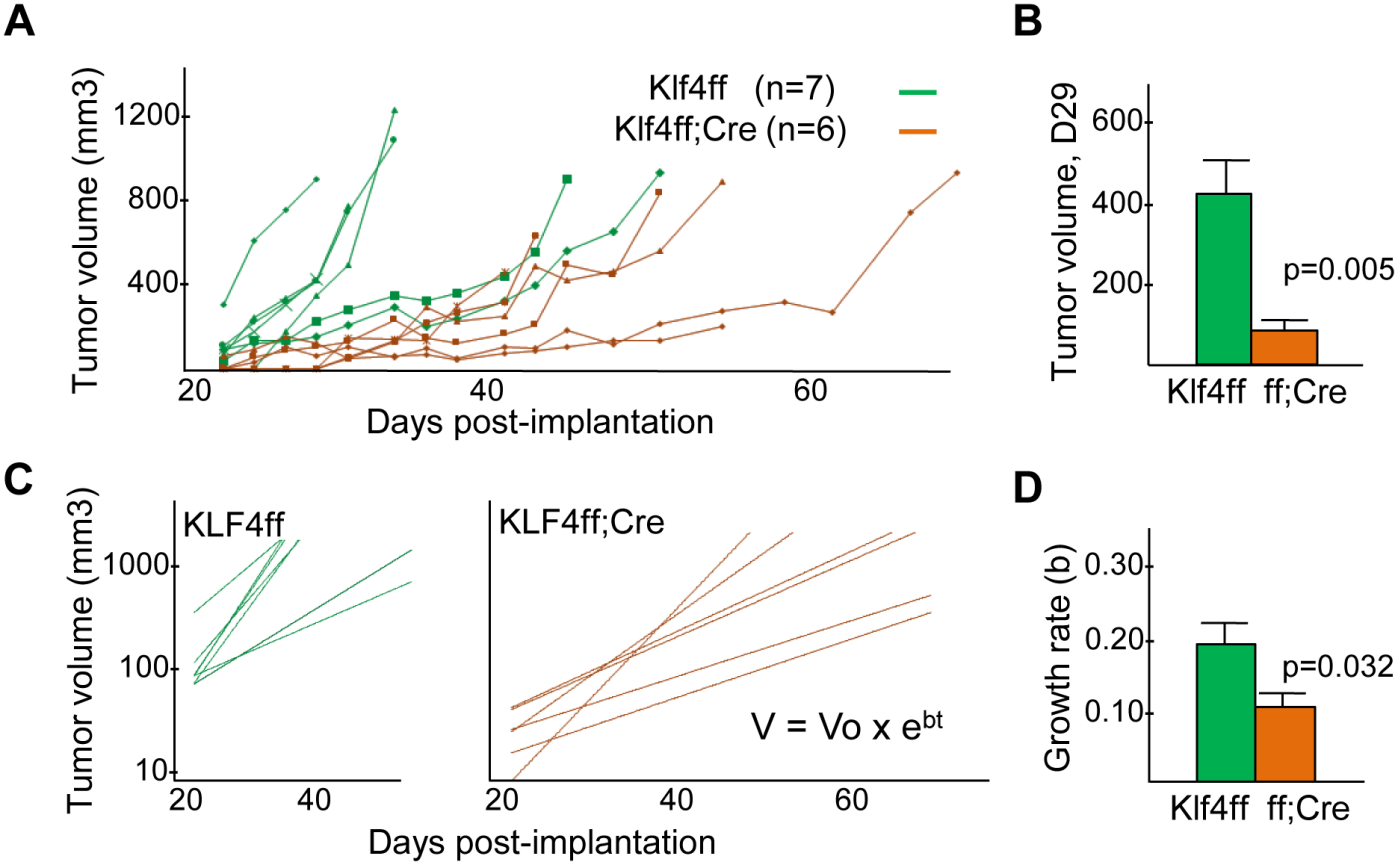
Hi-Myc prostate cancer grows more slowly in the absence of myeloid Klf4. **A)** Estimated tumor volumes evaluated every 2–3 days after inoculation of B6 Hi-Myc tumor cells SQ into the flanks of Klf4(f/f) or Klf4(f/f);Lys-Cre mice. **B)** Estimated day 29 tumor volumes (~Vo) (mean and SE). **C)** Best fit lines of estimated tumor volumes versus time, V = Vo + e^bt^ for these same data. **D)** Mean exponential gowth rates, “b”, of Hi-Myc tumors (mean and SE).

*Klf4* mRNA was reduced 11-fold in Klf4(f/f);Lys-Cre compared with Klf4(f/f) peritoneal macrophages (S2A Fig), consistent with prior findings with this model [19]. B6 Hi-Myc PCa cells were inoculated into the flanks of B6 Klf4(f/f) and Klf4(f/f);Lys-Cre mice and tumor volumes were monitored every 2–3 days beginning on day 22 (Fig 1A). Initial growth was uniformly slower in the absence of myeloid Klf4, with mean tumor volumes on day 29, with 6.4-fold lower mean tumor volumes in the Klf4(f/f);Lys-Cre recipients (Fig 1B). Plotting the estimated tumor volumes subsequent to day 22 on a logarithmic scale allows fitting later growth rates to best fit lines (Fig 1C), with the slopes of these lines on average ~2-fold lower in the Klf4(f/f);Lys-Cre cohort (Fig 1D). The average, estimated growth rates predict a 7-fold difference in tumor volume on day 29, similar to what was found by caliper measurements. In additional mice sacrificed on days 24–29 for tumor myeloid or T cell analyses, the tumor volumes were also significantly lower in the absence of myeloid Klf4 (S3 Fig).

### Hi-Myc prostate cancer has increased TAMs with reduced MR and increased CD11c in the absence of myeloid Klf4

**Fig 2:**
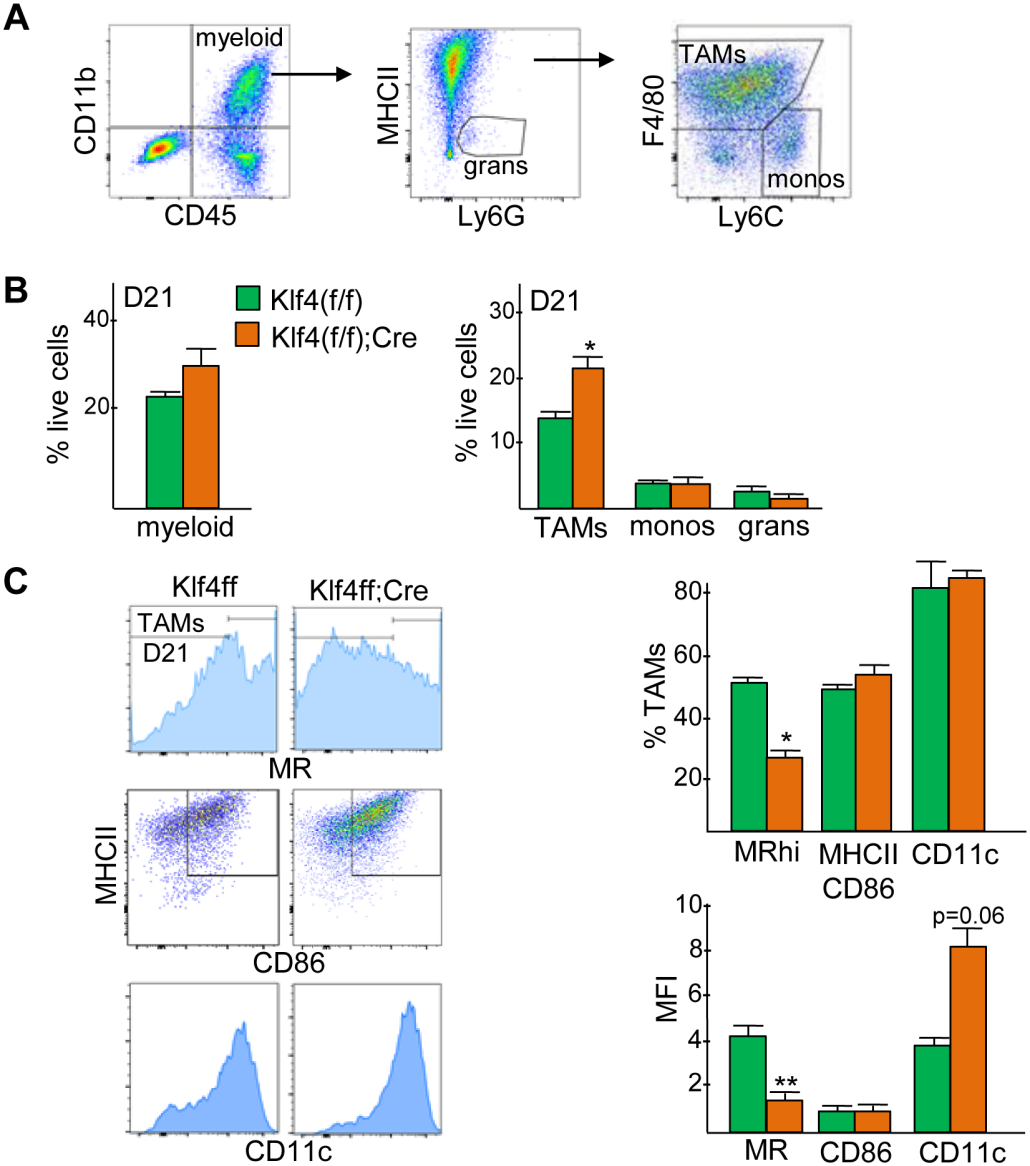
Hi-Myc prostate cancer increases TAMs with reduced mannose receptor in the absence of myeloid Klf4. **A)** Gating strategy for identifying CD45^+^CD11b^+^ tumor myeloid cells, and TAMs, monocytes, and granulocytes within the tumor myeloid population. **B)** Quantification of myeloid cells (left) and myeloid subsets (right) as a percentage of viable tumor cells in Klf4(f/f) and Klf4(f/f);Lys-Cre PCa recipients 21 days after inoculation of Hi-Myc PCa (mean and SE, n = 3). **C)** Representative FC plots (left) for MR, MHCII;CD86 and CD11c expression, bar graphs (right) showing the percentage of MR^hi^, MHCII^+^CD86^+^, and CD11c^+^ TAMs (top), and the mean fluorescence intensity (MFI) in these populations (bottom) on day 21 in Klf4(f/f) and Klf4(f/f);Lys-Cre PCa recipients (mean and SE, n = 3). * p <0.05, ** p < 0.01, *** p <0.001.

To evaluate how Klf4 influences myeloid composition and macrophage polarization in Hi-Myc tumors, we analyzed a panel of surface markers on dissociated tumor cells by multi-color flow cytometry (Fig 2A). CD45^+^CD11b^+^ myeloid cells represented ~20% of live tumor cells isolated on day 21 from Klf4(f/f) and ~29% of Klf4(f/f);Lys-Cre Hi-Myc PCa recipients, p = 0.07 (Fig 2B, right). The majority of tumor myeloid cells were F4/80^hi^Ly6C^lo/mid^ TAMs, and these increased approximately 1.5-fold in the absence of myeloid Klf4 (Fig 2B, left). The day 21 TAM population was further characterized by analysis of surface MR, MHCII/CD86, and CD11c (Fig 2C). MR^hi^ cells, representing M2-biased TAMs, were reduced 1.8-fold, and MHCII^+^CD86^+^ cells, representing M1-biased TAMs, were unchanged. The mean fluorescence intensity (MFI) of CD11c, present on activated macrophages [23], was increased 2-fold in Klf4(f/f);Lys-Cre compared with Klf4(f/f) PCa tumor recipients. These data demonstrate that myeloid Klf4 promotes an M2 TAM phenotype in Myc-driven prostate cancer.

### Hi-Myc prostate cancer growth and tumor myeloid cells are unaffected by absence of myeloid C/EBPβ

**Fig 3:**
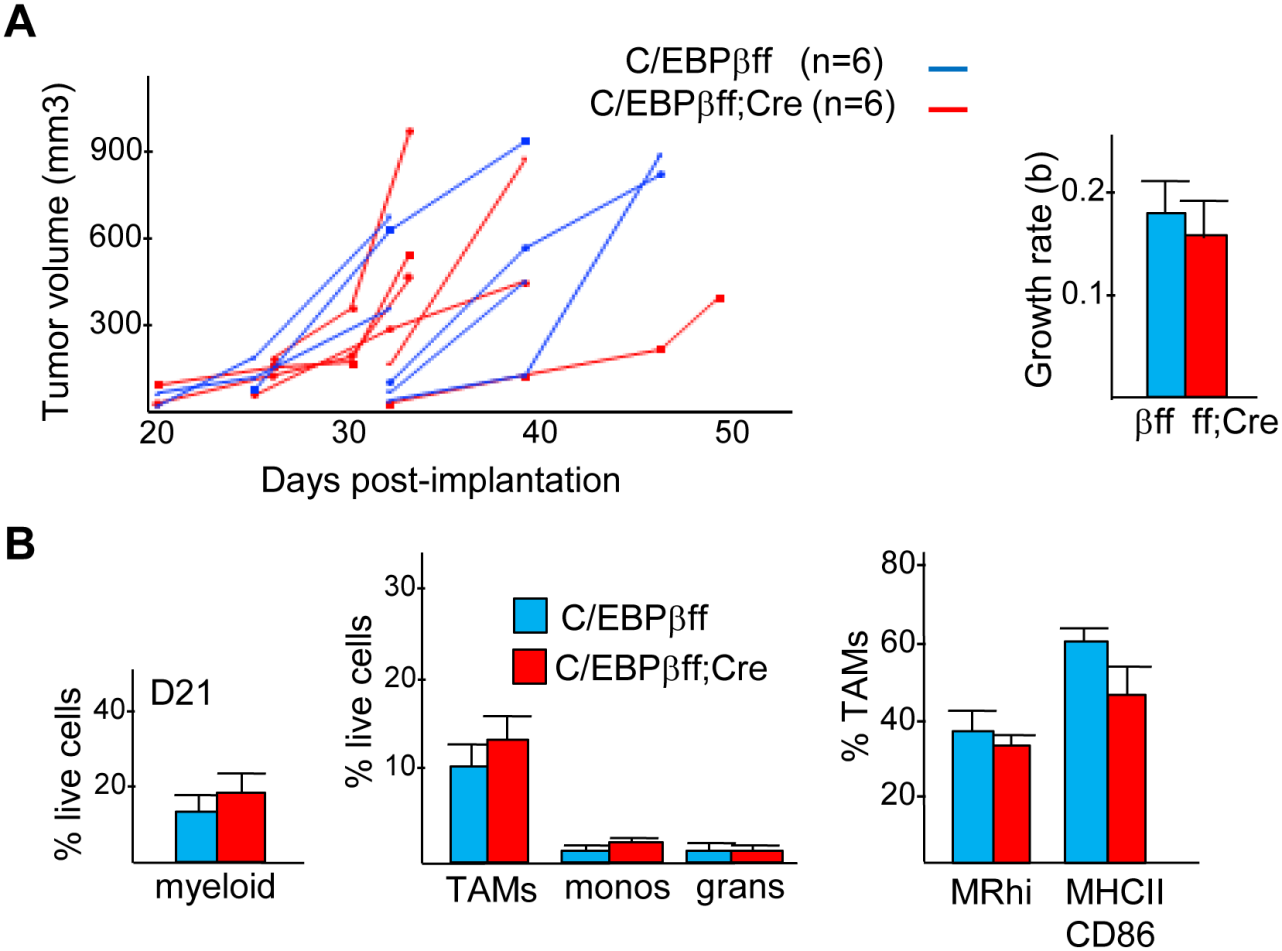
Absence of myeloid C/EBPβ does not affect growth of Hi-Myc prostate cancer. **A)** Estimated tumor volumes evaluated every 2–3 days after inoculation of B6 Hi-Myc PCa cells SQ into the flanks of C/EBPβ(f/f) or C/EBPβ(f/f);Lys-Cre mice. **B)** Percentage of myeloid cells, TAMs, monocytes, and granulocytes amongst viable tumor cells on day 21 (mean and SE, n = 3).

C/EBPβ is another transcription factor reported to promote M2 polarization [10]. To test whether tumor myeloid cells utilize C/EBPβ to drive an M2 phenotype in prostate cancer, we evaluated Hi-Myc tumors in C/EBPβ(f/f) and C/EBPβ(f/f);Lys-Cre mice. C/EBPβ mRNA was reduced >100-fold in C/EBPβ(f/f);Lys-Cre compared with C/EBPβ(f/f) peritoneal macrophages (S2B Fig). Hi-Myc PCa cells were inoculated into the flanks of B6 C/EBPβ(f/f) and C/EBPβ(f/f);Lys-Cre mice and tumor volumes were monitored every 2–3 days beginning on d21. Tumor growth rates were similar in the two cohorts (Fig 3A). Tumor cell FC analysis found no differences in the proportions of total myeloid cells, TAMs, monocytes, granulocytes, MR^hi^ TAMs, or MHCII^+^CD86^+^ TAMs on day 21 in control or C/EBPβ-deleted prostate cancer recipients (Fig 3B). Neither the proportion of TAMs expressing CD11c nor their CD11c MFI was affected by absence of C/EBPβ (not shown). These data demonstrate that myeloid expression of C/EBPβ does not affect TAM polarization or Hi-Myc tumor growth.

### Hi-Myc prostate cancer myeloid cells lacking Klf4 express RNAs associated with pro-inflammatory pathways

**Fig 4:**
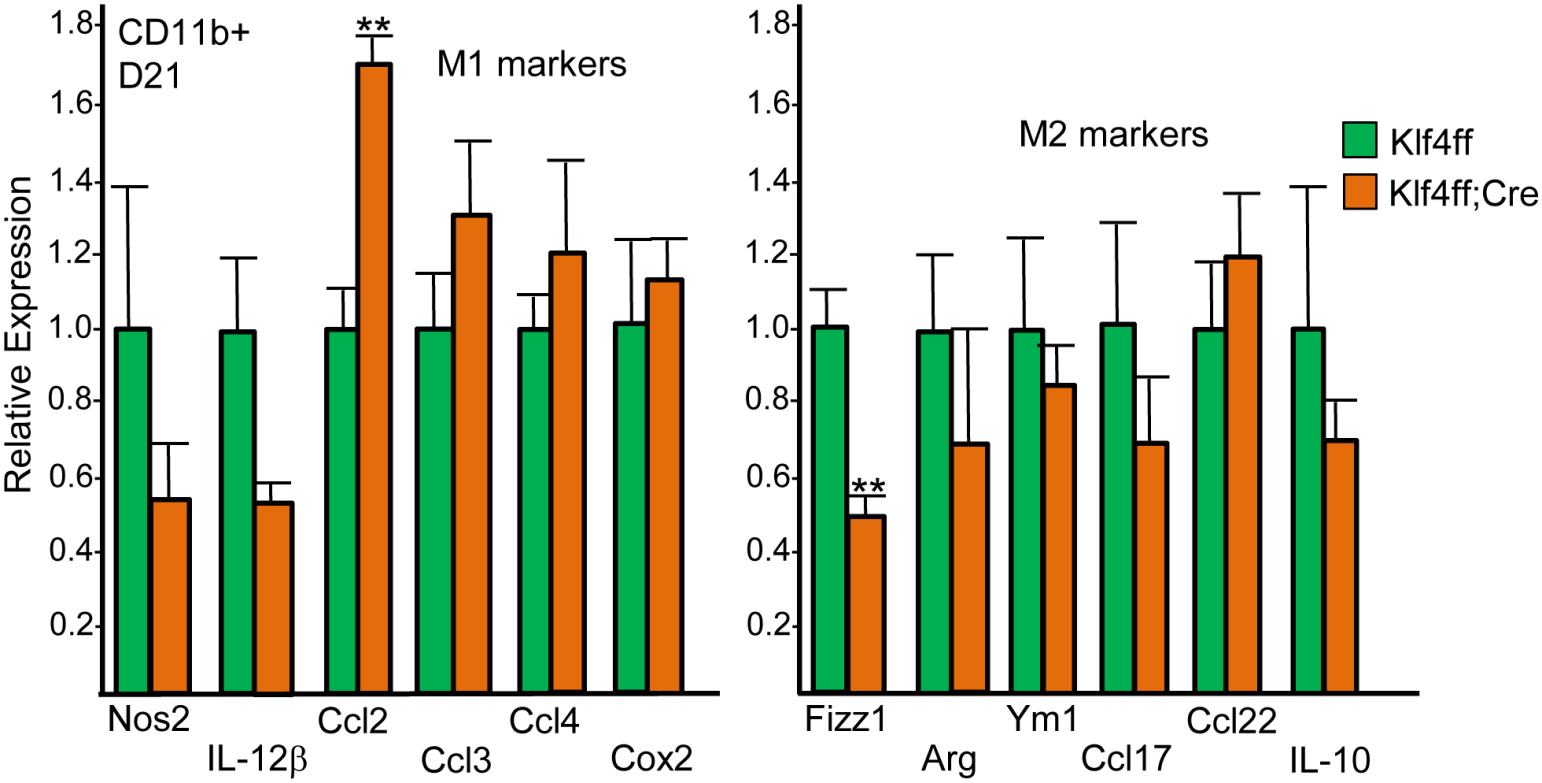
Expression of a subset of M1 and M2 mRNAs in Hi-Myc prostate cancer myeloid cells in Klf4(f/f) versus Klf4(f/f);Lys-Cre hosts. RNAs prepared from tumor CD11b^+^ cells on day 21 after Hi-Myc PCa inoculation were subjected to quantitative RT-PCR analysis for the indicated M1 and M2 markers and for the RNA encoding *cyclophilin A* as an internal control. The relative expression of each mRNA is shown for Klf4(f/f) and Klf4(f/f);Lys-Cre recipients, with expression in Klf4(f/f) mice set to 1.0 (mean and SE, n = 5).

CD11b^+^ myeloid cells were isolated from Hi-Myc prostate cancers 21 days after inoculation into Klf4(f/f) or Klf4(f/f);Lys-Cre recipients. RNA from these cells was analyzed for a panel of M1 and M2 macrophage markers by qRT-PCR (Fig 4). Most were not significantly changed, though the M1 marker *Ccl2* was increased and the M2 marker *Fizz1* decreased in the absence of myeloid Klf4. *IL-1b* and *IL-6* were also not significantly different between the two groups (not shown). Consistent with our findings with peritoneal macrophages, TAM *Klf4* mRNA was reduced 10-fold in the presence of Lys-Cre (S4 Fig).

To further evaluate the influence of Klf4 on TAM polarization, RNAs isolated from CD11b^+^ tumor myeloid cells from two tumors in each host were subjected to global gene expression analysis. mRNAs corresponding to 277 genes were up-regulated and 119 down-regulated 1.4-fold or greater in the absence of myeloid Klf4 (S2 Table). Ingenuity Pathway analysis revealed activation of pathways involved in cellular movement, inflammation and adhesion. Genes increased or decreased within these pathways in our data set are listed (Table 1). Because “Atherosclerosis Signaling” was the top regulated pathway from our analysis we compared our microarray data to a recent proteomic analysis of atherosclerotic plaques from human patients that underwent endarterectomy [24]. 20 genes that were differentially regulated in CD11b^+^ cells overlapped with 146 extracellular matrix (ECM) or ECM-related proteins identified in atherogenic plaques. Of these 20 genes, 19 were upregulated in tumors that formed in Lys-Cre;Klf4(f/f) mice (Table 2). These results suggest that myeloid deletion of Klf4 promotes an atherogenic-like microenvironment in Hi-Myc prostate cancer.

**Table 1:** Ingenuity pathway analysis of Klf4(f/f) vs Klf4(f/f);Lys-Cre PCa myeloid cells*.

Pathway (-logB-H p-value)	RNAs higher in Klf4(f/f);Lys-Cre	RNAs higher in Klf4(f/f)
Atherosclerosis Signaling (5.01)	Mmp3, CD36, Clu, Cma1, Col1a1, Col1a2, Col3a1, IL1rn, Rbp4, S100a8	Lyz, Pdgfb, Tnf
LXR/RXR Activation (4.56)	Ahsg, CD36, Clu, IL1r2, IL1rn, Lbp, Rbp4, Vtn, S100a8	Lyz, Nos2,Tnf
Granulocyte Adhesion and Diapedesis (3.98)	Ccl7, Cldn3, Cldn10, Fpr1, Hspb1 IL1r2, IL1rn, Mmp3, Pf4, Sell	Ccl24,Cxcl9,Tnf
Agranulocyte Adhesion and Diapedesis (3.79)	Acta2, Ccl7, Cldn3, Cldn10, IL1rn, Mmp3, Myl9, Pf4, Sell	Ccl24, Cxcl9, Fn1, Tnf
Hepatic Fibrosis/Hepatic Stellate Cell Activation (3.52)	Acta2, Ccr7, Col1a1, Col1a2, Col3a1, Igfbp5, IL1r2, Lbp, Myl9	Fn1, Pdgfb, Tnf
Acute Phase Response Signaling (2.67)	Ahsg, C1r, Hp, IL1rn, Lbp, Rbp1, Rbp4, Saa3, Serping1	Fn1, Tnf
Altered T Cell and B Cell Signaling in Rheumatoid Arthritis (RA) (2.67)	Spp1, IL1rn	HLA-DMA, HLA-DQA1, HLA-DRB5, Tlr12, Tnf, Tnfsf13b
Role of Macrophages, Fibroblasts and Endothelial Cells in RA (2.49)	Dkk3, IL1r2, IL1rn, Mmp3, Sfrp2, Traf4	Camk2d, Cebpe, Fn1, Nos2, Pdgfb, Prkcb, Tlr12, Tnf, Tnfsf13b
T Helper Cell Differentiation (2.49)		Icosl, IL2ra, HLA-DMA, HLA-DQA1, HLA-DRB5, Tgfbr1, Tnf
Communication Between Innate and Adaptive Immune Cells (2.46)	Ccr7, IL1rn	HLA-DRB5, Ifnb1 Tlr12, Tnf, Tnfsf13b

*Top 10 Ingenuity Pathways comparing mean RNA levels from CD11b^+^ myeloid cells isolated from Hi-Myc PCa tumors in Klf4(f/f) vs Klf4(f/f)Lys-Cre hosts (n = 2 per group).

**Table 2:** Extracellular atherosclerosis plaque proteins in myeloid cells lacking Klf4*.

RNAs higher in Klf4(f/f);Lys-Cre	RNAs higher in Klf4(f/f)
Aebp1, Clu, Col1a1, Col3a1, Cpa3, Dcn, Htra1, Lama5, Lamb2, Lum, Mfge8, Pcolce, S100a8, S100a9, Saa3, Serping1, Spp1, Tnxb, Vtn	Fn1

*RNAs increased or reduced >1.4-fold that correspond to extracellular proteins in carotid plaques but not in normal carotid arteries.

### Absence of myeloid Klf4 increases activated CD8^+^ T-cells in Hi-Myc prostate cancer

**Fig 5:**
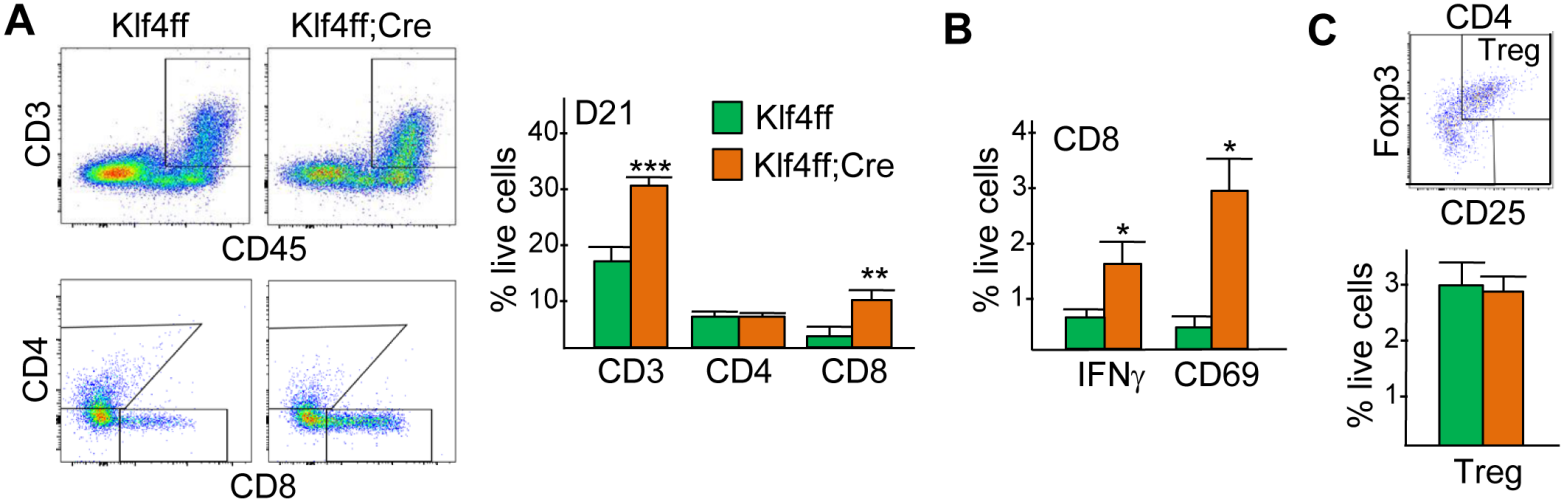
Increased number of activated CD8 T cells in Hi-Myc prostate cancer in hosts lacking myeloid Klf4. **A)** Representative FC plots for CD45;CD3 expression and for CD4 and CD8 expression amongst CD45^+^CD3^+^ tumor cells (left) and the percentage of CD3, CD4, or CD8 T cells amongst viable tumor cells on day 21 in Klf4(f/f) and Klf4(f/f);Lys-Cre Hi-Myc PCa recipients (right, mean and SE, n = 3). **B)** Percentage of CD45^+^CD3^+^CD8^+^IFNγ^+^ or CD45^+^CD3^+^CD8^+^CD69^+^ cells amongst live tumor cells on d21. **C)** Percentage CD3^+^CD4^+^CD25^+^Foxp3^+^ Treg cells on day 21 in Klf4(f/f) and Klf4(f/f);Lys-Cre PCa recipients (right, mean and SE, n = 3).

Induction of pro-inflammatory pathways in tumor myeloid cells by Klf4 deletion might induce immune cell influx and activation. Prostate cancers from Klf4(f/f) or Klf4(f/f);Lys-Cre mice were analyzed for CD3, CD4 and CD8 tumor-infiltrating lymphocytes (TIL) on day 21 (Fig 5A). CD3^+^ cells, representing total T cells, were increased 2-fold, and CD8 TIL were increased 4-fold in the absence of myeloid Klf4, whereas CD4 T cell numbers were unchanged. In absolute terms, CD8 T cells represented ~8% of viable tumor cells. Activation of CD8 T cells was assessed by staining for intracellular IFNγ or surface CD69 (Fig 5B). The frequency of CD8 T cells expressing these markers were markedly increased in Klf4(f/f);Lys-Cre hosts, representing 1.5% or 3% of viable tumor cells. The proportion of CD3^+^CD4^+^CD25^+^Foxp3^+^ Tregs was not changed (Fig 5C), indicating an overall increase in the CD8:Treg ratio.

**Fig 6:**
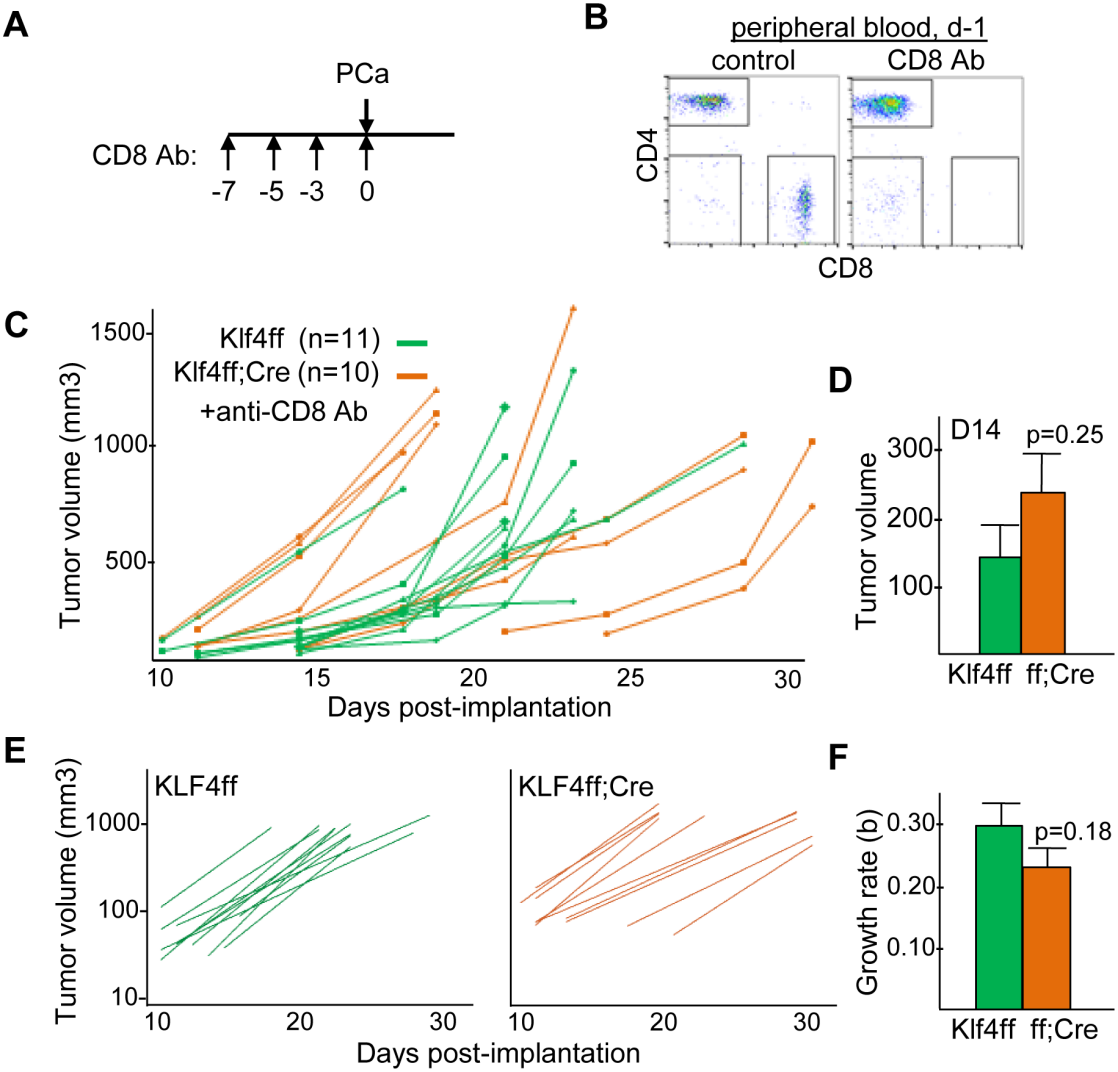
CD8 T cell depletion eliminates slower growth of Hi-Myc prostate cancer in mice lacking myeloid Klf4. **A)** Klf4(f/f) and Klf4(f/f);Lys-Cre mice received CD8 antibody (Ab) on days -7, -5, -3, and 0 followed by Hi-Myc PCa cell inoculation on d0, as diagrammed. **B)** Representative CD4;CD8 FACS plots demonstrating efficient depletion of CD8 T cells from the peripheral blood one day prior to PCa inoculation **C)** Estimated tumor volumes evaluated every 2–3 days after inoculation. **D)** Estimated day 14 tumor volumes, Vo (mean and SE). **E)** Best fit lines for the estimated tumor volumes versus time, V = Vo + e^bt^ for these same data. **F)** Mean exponential growth rates, “b”, of Hi-Myc tumors (mean and SE).

To determine the contribution of CD8 T cells to the slower growth of Hi-Myc PCa calls in Klf4(f/f);Lys-Cre mice, we used antibody to deplete CD8 T cells. Klf4(f/f) and Klf4(f/f);Lys-Cre mice received 4 doses of CD8 antibody 7, 5, 3 and 0 days prior to PCa cell implantation, and depletion was confirmed by FC analysis of peripheral blood 1 day prior to implantation (Fig 6A and 6B). Hi-Myc PCa growth rates were similar in both cohorts (Fig 6C–6E). Thus, CD8 depletion eliminated the difference in growth rates between Hi-Myc tumors grown in Klf4 (f/f) and Klf4(f/f); Lys-Cre mice shown in Fig 1. These results suggest that CD8 T cells are critical for suppressing Hi-Myc prostate tumor growth in Klf4(f/f); Lys-Cre mice.

## Discussion

Klf4 and C/EBPβ are transcription factors that play a role in monocyte development and favor macrophage M2 polarization. The Lys-Cre transgene deletes floxed alleles in macrophages, activated monocytes, and granulocytes [25]. We found that B6 Hi-Myc PCa tumors grew more slowly when inoculated subcutaneously in Klf4(f/f);Lys-Cre compared with Klf4(f/f) hosts, both during the first 21–29 days when tumors become detectable and thereafter. In contrast, there was no difference in Hi-Myc PCa growth kinetics in C/EBPβ(f/f);Lys-Cre versus C/EBPβ(f/f) recipients. Limitations of our study include lack of evaluation in the orthotopic prostate environment, which can be challenging due to early ureteral obstruction and difficulty with monitoring tumor growth, and lack of evaluation of the effects of myeloid KLF4 deletion using additional prostate cancer models.

Amongst tumor myeloid cells, TAMs predominated over monocytes and granulocytes in this model, and F4/80^+^ TAMs were increased 2-fold in the absence of Klf4. TAMs lacking Klf4 manifested substantial reduction in surface MR and *Fizz1* mRNA expression (M2 markers) and a gene expression signature demonstrating activation of multiple pro-inflammatory pathways. CD11b^+^ cells from tumors grown in Klf4(f/f);Lys-Cre mice displayed increased expression of several interferon-regulated genes (*Ifi27l2a*, *Isg20*, *Oasl1*, *Irf7* and *Mx2*), indicating an activated innate immune cell phenotype. However, several classic M1 genes, including *Ifnb*, *Tnf*, *Cxcl9*, and genes encoding MHC II subunits showed higher expression in Klf4(f/f) control mice. This suggests that deletion of Klf4 influences specific aspects of M1 differentiation and represses others in the Hi-Myc PCa microenvironment and perhaps less than that seen upon culture of Klf4(f/f) versus Klf4(f/f);Lys-Cre BMDM in IFNγ (M1 inducer) or IL-4 (M2 inducer) [19].

Notably, “Atherosclerosis Signaling” was the top pathway associated with the gene expression pattern in Klf4-deleted CD11b^+^ myeloid cells isolated from Hi-Myc tumors. Atherosclerosis is a progressive disease that narrows the vascular lumen through a buildup of arterial plaque, an amalgam of lipids, macrophages and other leukocytes, smooth muscle cells, endothelial cells (EC), necrotic regions, and extracellular proteins. Nascent atherosclerotic plaques activate the vascular endothelium by increasing shear stress and by releasing pro-inflammatory cytokines, promoting recruitment and invasion of monocytes and other blood cells [26,27]. Klf4 has been identified as a critical molecule involved in the pathogenesis of atherosclerosis in macrophages and ECs. Exposure of peritoneal macrophages to an inflammatory lipid reduces Klf4 and increases M1 mRNAs, the latter induced to a greater extent in Klf4(f/f);Lys-Cre macrophage, and absence of myeloid Klf4 augments atherosclerosis in ApoE-/- mice [28]. Exposure of ECs to pro-inflammatory cytokines or shear stress reduces their expression of Klf4, deletion of endothelial Klf4 augments atherosclerosis, and transgenic EC-Klf4 reduces atherosclerosis [29,30]. Notably, we found that 20 differentially regulated genes from our microarray analysis matched proteomic analysis of extracellular proteins from atherosclerotic plaques [24], with a striking 19 out of these 20 mRNAs having higher expression in myeloid cells obtained from prostate cancers growing in Lys-Cre;Klf4(f/f) compared with the Klf4(f/f) hosts.

In addition, we observed a nearly 2-fold increase in the frequency of tumor CD3^+^ T cells and a 4-fold increase in tumor CD8^+^ T cells in response to myeloid Klf4 deletion. Amongst CD8 T-cells, there was no change in the percentage of cells expressing IFNγ or CD69, indicating that myeloid Klf4 deletion did not augment CD8 lymphocyte activation. We found that CD8 T cells were critical for the growth defect caused by absence of myeloid Klf4 because depletion of CD8 T cells eliminated the growth deficit observed in Hi-Myc tumors. As Treg numbers were unchanged, the CD8 effector:Treg ratio was similarly increased. Notably, the CD8:Treg ratio has been shown to correlate with favorable prognosis in prostate cancer [31,32]. Cxcl9, a widely reported T-cell chemoattractant, was down-regulated in Klf4(f/f);Lys-Cre mice and our data did not reveal up-regulation of genes involved in T cell chemotaxis. These findings suggest that upregulation of chemokines is not likely to explain the rise in tumor T cell frequency in the Klf4(f/f);Lys-Cre mice. In co-cultures of Klf4(f/f) or Klf4(f/f);Lys-Cre CD11b^+^ tumor cells with splenic T cells stimulated with anti-CD3 and anti-CD28, we did not observe a difference in CD8 T cell proliferation (not shown), suggesting that increased proliferation also does not explain the observed increase in T cell frequency.

Increased T cell numbers in Hi-Myc tumors forming in Klf4(f/f);Lys-Cre mice is potentially related to a change in the composition of the ECM generated by CD11b^+^ tumor myeloid cells lacking Klf4. The expression of α_5_, β_1_ and β_3_ integrins promotes retention and motility of activated T cells in inflamed non-lymphoid tissues dependent upon binding to fibronectin and collagenous fibers of the ECM [33–36]. In atherosclerotic lesions, T cells accumulate within the fibrous cap, where the ECM is composed of types I and III collagen with small patches of fibronectin [37]. CD8 T cells enter the arterial wall of atherogenic plaques and represent the dominant lymphocyte during advanced, high risk plaques, which coincides with active degradation of the plaque ECM by infiltrating macrophages [38,39]. We observed an increase in types I and III collagen, *Pcolce* (a collagen endopeptidase required for maturation of pro-collagen), and *Dcn* (a proteoglycan involved in the staggered assembly of collagen molecules in myeloid cells from Klf4(f/f);Lys-Cre vs Klf4(f/f) tumors. Low density ECM has the potential to suppress tumorigenicity via T cell recruitment; for example, in explants from human lung tumors and lung tumor xenografts T cells are motile along low density ECM fibers and excluded from areas of high ECM density [40,41]. Interestingly, myeloid *Fizz1/Relmα* expression, which was substantially decreased in Klf4(f/f);Lys-Cre tumor myeloid cells, was shown to promote collagen-cross linking, increase collagen fibril density and facilitate wound healing in a model of cutaneous injury [42], further suggesting presence of low-density collagen fibers in Hi-Myc PCa tumors in Klf4(f/f);Lys-Cre recipients.

In conclusion, our findings implicate myeloid Klf4 in prostate cancer progression. Targeting myeloid Klf4 may provide therapeutic benefit to PCa patients via induction of a pro-inflammatory myeloid program and increased density of activated CD8 T cells and CD8:Treg ratio, potentially through effects on macrophage-mediated ECM remodeling.

## Supporting information

S1 Fig Hi-Myc prostate cancer cells. A tumor grown subcutaneously in a WT host was fixed, paraffin-embedded, sectioned, and subjected to hematoxylin-eosin (H/E) staining or to immunohistochemistry for c-Myc or androgen receptor (AR). Images shown are 60X.
(TIF)

S2 Fig Markedly reduced expression of floxed *Klf4* or *Cebpb* in macrophages mediated by Lys-Cre. **A)** RNA isolated from peritoneal macrophages from Klf4(f/f) or Klf4(f/f);Lys-Cre mice were subjected to quantitative RT-PCR for Klf4 relative to *cyclophilin A* (n = 2/group) **B)** RNA isolated from peritoneal macrophages from C/EBPβ(f/f) or C/EBPβ(f/f);Lys-Cre mice were subjected to quantitative RT-PCR for *Cebpb* relative to *cyclophilin A* (n = 2/group).
(TIF)

S3 Fig Absence of myeloid Klf4 reduces Hi-Myc prostate cancer tumor volumes in additional recipients. Tumor volumes assessed at the time of sacrifice of mice used for tumor myeloid or T cell analysis are shown after inoculation into Klf4(f/f) or Klf4(f/f);Lys-Cre recipients.
(TIF)

S4 Fig Marked reduction of Klf4 in Hi-Myc prostate cancer tumors in Klf4(f/f);Lys-Cre mice. RNAs prepared from tumor CD11b^+^ cells on day 21 after Hi-Myc PCa inoculation were subjected to quantitative RT-PCR analysis for *Klf4* and for the RNA encoding *cyclophilin A* as an internal control.
(TIF)

S1 Table Primers used for quantitative RT-PCR. (DOCX)

S2 Table RNAs whose expression was changed >1.4-fold by myeloid Klf4 deletion in CD11b^+^ prostate cancer tumor cells. (XLSX)
