# Supplementary figures and images for: Absence of myeloid Klf4 reduces prostate cancer growth with pro-atherosclerotic activation of tumor myeloid cells and infiltration of CD8 T cells
Source: PLoS One. 2018 Jan 11;13(1):e0191188. doi: 10.1371/journal.pone.0191188 (PMC5764416; doi:10.1371/journal.pone.0191188)

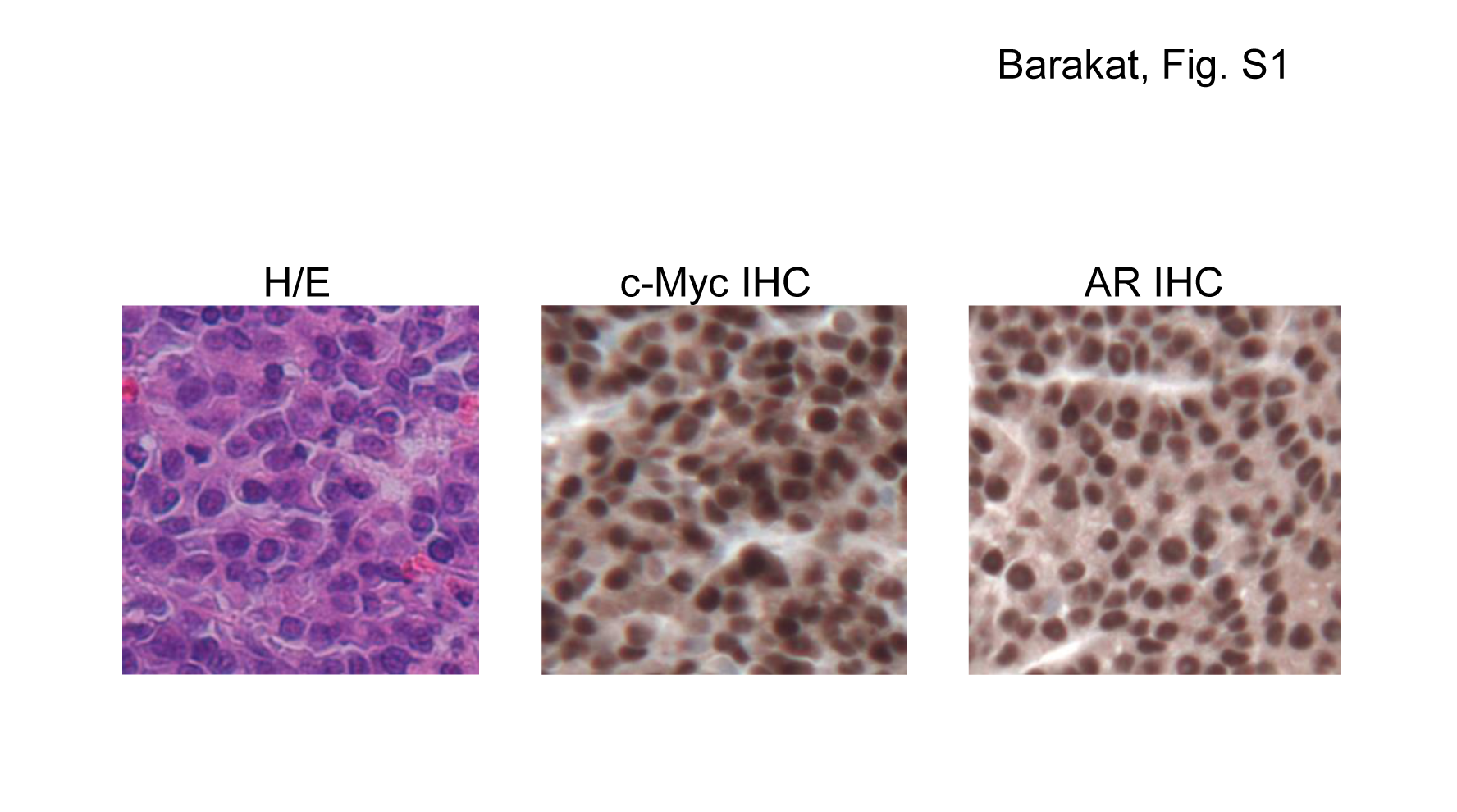

Supplement: S1 Fig — A tumor grown subcutaneously in a WT host was fixed, paraffin-embedded, sectioned, and subjected to hematoxylin-eosin (H/E) staining or to immunohistochemistry for c-Myc or androgen receptor (AR). Images shown are 60X. (TIF) [file pone.0191188.s001.tif]

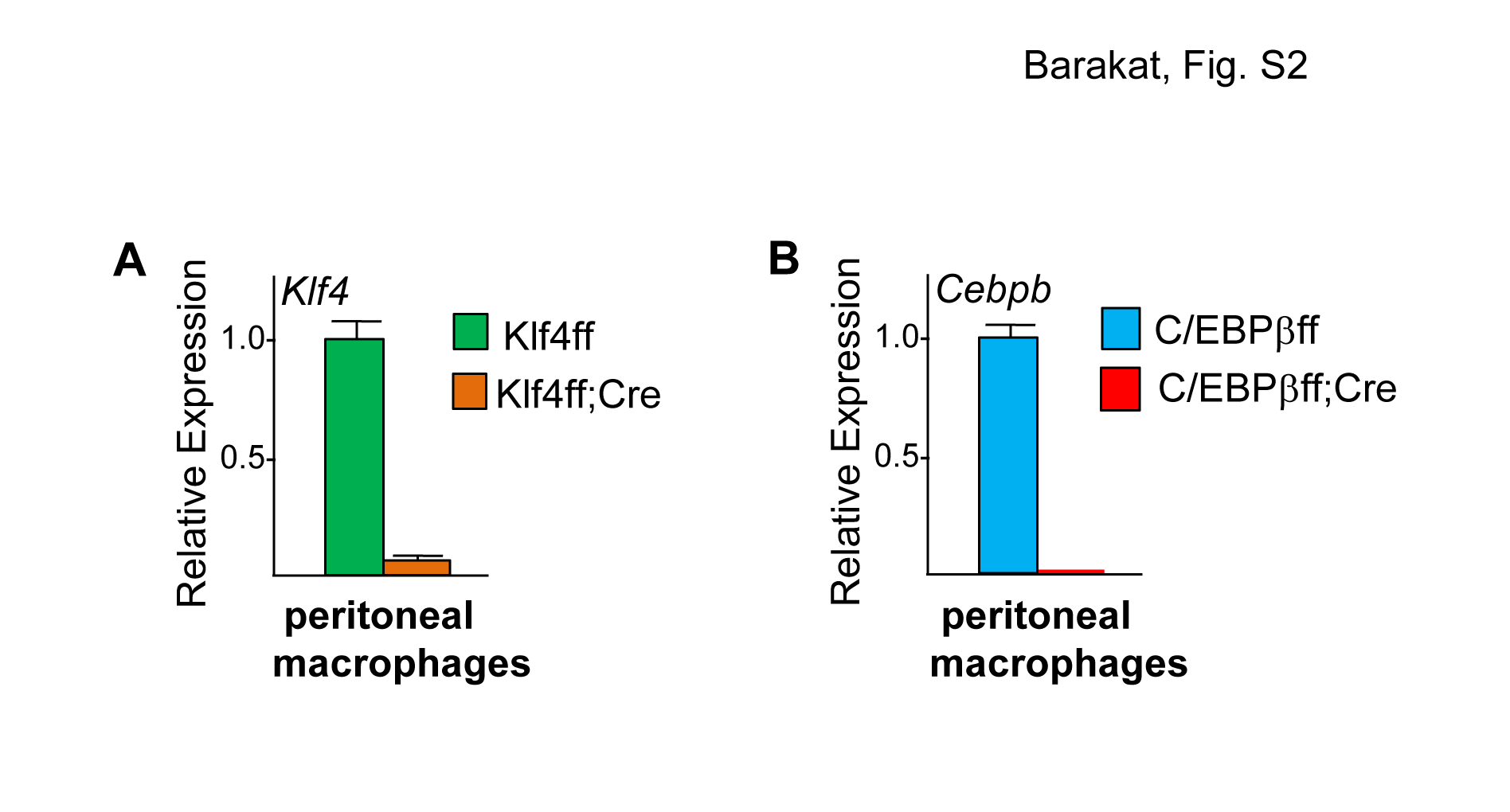

Supplement: S2 Fig — A) RNA isolated from peritoneal macrophages from Klf4(f/f) or Klf4(f/f);Lys-Cre mice were subjected to quantitative RT-PCR for Klf4 relative to cyclophilin A (n = 2/group) B) RNA isolated from peritoneal macrophages from C/EBPβ(f/f) or C/EBPβ(f/f);Lys-Cre mice were subjected to quantitative RT-PCR for Cebpb relative to cyclophilin A (n = 2/group). (TIF) [file pone.0191188.s002.tif]

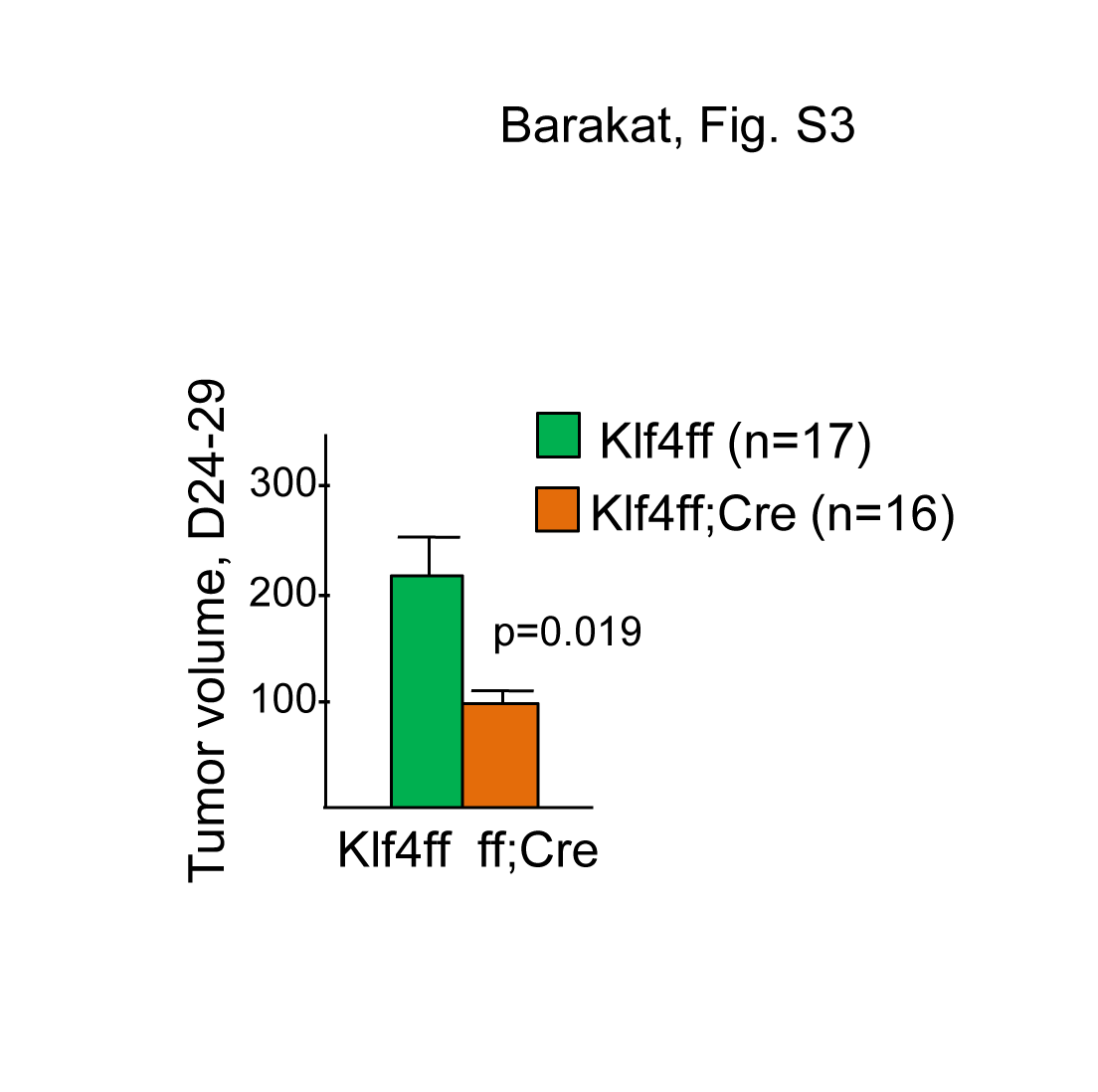

Supplement: S3 Fig — Tumor volumes assessed at the time of sacrifice of mice used for tumor myeloid or T cell analysis are shown after inoculation into Klf4(f/f) or Klf4(f/f);Lys-Cre recipients. (TIF) [file pone.0191188.s003.tif]

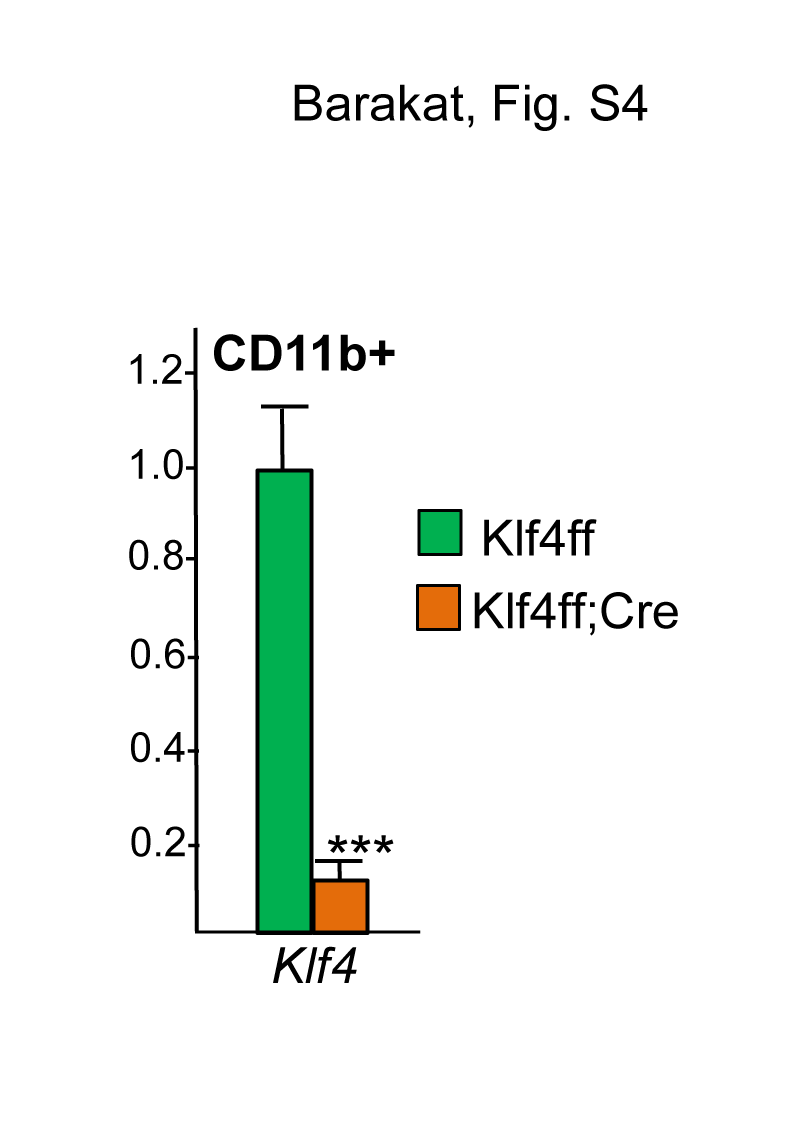

Supplement: S4 Fig — RNAs prepared from tumor CD11b+ cells on day 21 after Hi-Myc PCa inoculation were subjected to quantitative RT-PCR analysis for Klf4 and for the RNA encoding cyclophilin A as an internal control. (TIF) [file pone.0191188.s004.tif]
