# Supplementary material for: Absence of myeloid Klf4 reduces prostate cancer growth with pro-atherosclerotic activation of tumor myeloid cells and infiltration of CD8 T cells
Source: PLoS One. 2018 Jan 11;13(1):e0191188. doi: 10.1371/journal.pone.0191188 (PMC5764416; doi:10.1371/journal.pone.0191188)
Supplement: S1 Table — (DOCX) [file pone.0191188.s005.docx]

**Table S1. RT-PCR primer pairs**

| *Arg* | F: TACAAGACAGGGCTCCTTTCAG  R: TGAGTTCCGAAGCAAGCCAA |
| --- | --- |
| *Ccl17* | F: CGAGAGTGCTGCCTGGATTA  R: CCTGGACAGTCAGAAACACGAT |
| *Ccl22* | F: GCCAGGACTACATCCGTCAC  R: TGACGGTTATCAAAACAACGCC |
| *Cyclophilin A* | F: GAGCTGTTTGCAGACAAAGTTC  R: CCCTGGCACATGAATCCTGG |
| *Fizz1* | F: TGCCAATCCAGCTAACTATCCC  R: ACGAGTAAGCACAGGCAGTT |
| *Ym1* | F: CCAGCAGAAGCTCTCCAGAAG  R: TGGTAGGAAGATCCCAGCTGTA |
| *IL-1β* | F: GCAGAGTTCCCCAACTGGTA  R: GGTTTCTTGTGACCCTGAGC |
| *IL-6* | F: AGTCCGGAGAGGAGACTTCA  R: TTGCCATTGCACAACTCTTT |
| *IL-10* | F: GCCGGGAAGACAATAACTGC  R: GGCAACCCAAGTAACCCTTAAA |
| *IL-12β* | F: GGAGGGGTGTAACCAGAAAGG  R: GAGCTTGCACGCAGACATTC |
| *Ccl2* | F: ACCAGCCAACTCTCACTGAA  R: GCTGGTGAATGAGTAGCAGCA |
| *Ccl3* | F: CCCTCTGTCACCTGCTCAAC  R: CGTGGAATCTTCCGGCTGTA |
| *Ccl4* | F: TATGAGACCAGCAGTCTTTGCT  R: TGCTCAGTTCAACTCCAAGTCA |
| *Nos2* | F: AGACCTCAACAGAGCCCTCA  R: TCGAAGGTGAGCTGAACGAG |
| *Cox2* | F: GGGCCATGGAGTGGACTTAAA  R: TGCAGGTTCTCAGGGATGTG |
| *Tnfα* | F: CCAAAGGGATGAGAAGTTCC  R: CTCCACTTGGTGGTTTGCTA |
| *Klf4* | F: GGCCCAACTACCCTCCTTTC  R: TAGTCACAAGTGTGGGTGGC |
| *Cebpb* | F: GACAAGCTGAGCGACGAGTA  R: GCTTGAACAAGTTCCGCAGG |
